# Multianalytical Approach to Understand Polyphenol-Mal d 1 Interactions to Predict Their Impact on the Allergenic Potential of Apples

**DOI:** 10.1021/acs.jafc.4c01555

**Published:** 2024-07-11

**Authors:** Julia. A H Kaeswurm, Birgit Claasen, Pia. S Mayer, Maria Buchweitz

**Affiliations:** † Department of Chemistry, University Hamburg, Institute of Food Chemistry, Martin-Luther-King-Platz 6, 20146 Hamburg, Germany; ‡ Department of Food Chemistry, University Stuttgart, Institute of Biochemistry and Technical Biochemistry, Allmandring 5b, 70569 Stuttgart, Germany; § University Stuttgart, Institute of Organic Chemistry, Pfaffenwaldring 55, 70569 Stuttgart, Germany

**Keywords:** isothermal titration calorimetry, ^1^H−^15^N-HSQC NMR, mass spectrometry, polyphenol–protein interactions, apple allergy, release, in vitro oral digestion

## Abstract

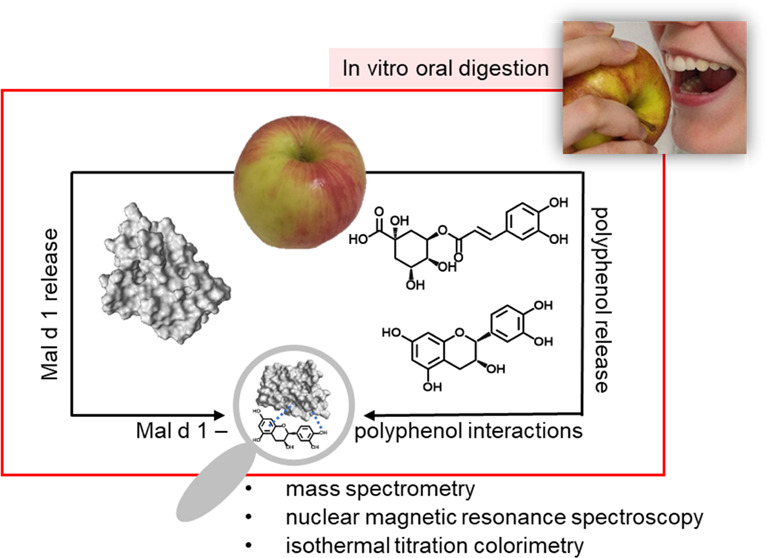

Interactions between phenolic compounds and the allergen Mal d 1 are discussed to be the reason for better tolerance of apple cultivars, which are rich in polyphenols. Because Mal d 1 is susceptible to proteolytic digestion and allergenic symptoms are usually restricted to the mouth and throat area, the release of native Mal d 1 during the oral phase is of particular interest. Therefore, we studied the release of Mal d 1 under different in vitro oral digestion conditions and revealed that only 6–15% of the total Mal d 1 present in apples is released. To investigate proposed polyphenol-Mal d 1 interactions, various analytical methods, e.g., isothermal titration calorimetry, ^1^H–^15^N-HSQC NMR, and untargeted mass spectrometry, were applied. For monomeric polyphenols, only limited noncovalent interactions were observed, whereas oligomeric polyphenols and browning products caused aggregation. While covalent modifications were not detectable in apple samples, a Michael addition of epicatechin at cysteine 107 in r-Mal d 1.01 was observed.

## 1 Introduction

Apples (*Malus domestica* Borkh.) are a rich source of vitamins, fibers, and bioactive compounds, in particular polyphenols (PP), which are known for various health benefits.^1^ However, people can also suffer from allergies against apples. In Southern Europe, patients are commonly sensitized to the nonspecific lipid transfer protein Mal d 3, which can cause severe symptoms upon apple consumption. In contrast, apple allergy in Northern and Central Europe, is caused by the homology in conformation of the pathogenesis-related class 10 (PR-10) proteins Mal d 1, from apples, and Bet v 1, from birch,^2^ leading to the development of a cross allergic reaction in individuals who are allergic to birch pollens. It is estimated that in Germany, over 2 million people are affected.^3^ Symptoms manifest as swelling, burning, or itching of the mouth, lips, and tongue, or a perceived tightness of the throat.^4^ As the conformational epitopes, triggering the allergic reaction, are degraded during gastrointestinal digestion, symptoms are usually restricted to the oral cavity. Even though the symptoms are usually mild, affected persons often avoid the consumption of fresh apples.

Consumer surveys and clinical studies have indicated apple variety-specific differences in the allergenic potential.^5−7^ Especially “old” varieties grown locally in traditional orchard meadows are reported as being better tolerable than “new” varieties of commercial importance, such as Golden Delicious. As the origin dates of these “new” breeds might be similar to the “old” varieties, these terms are misleading, and therefore, we brand these two groups as “traditional” and “commercial”.^8^ An impact of the Mal d 1 content and isoallergen profile on the allergenic potential of an apple variety is assumed.^9,10^ However, since traditional varieties are often characterized by higher phenolic contents than commercial breeds,^11−13^ an effect of PP on the allergenicity is discussed in the literature.^3,9,14^

**Figure 1:**
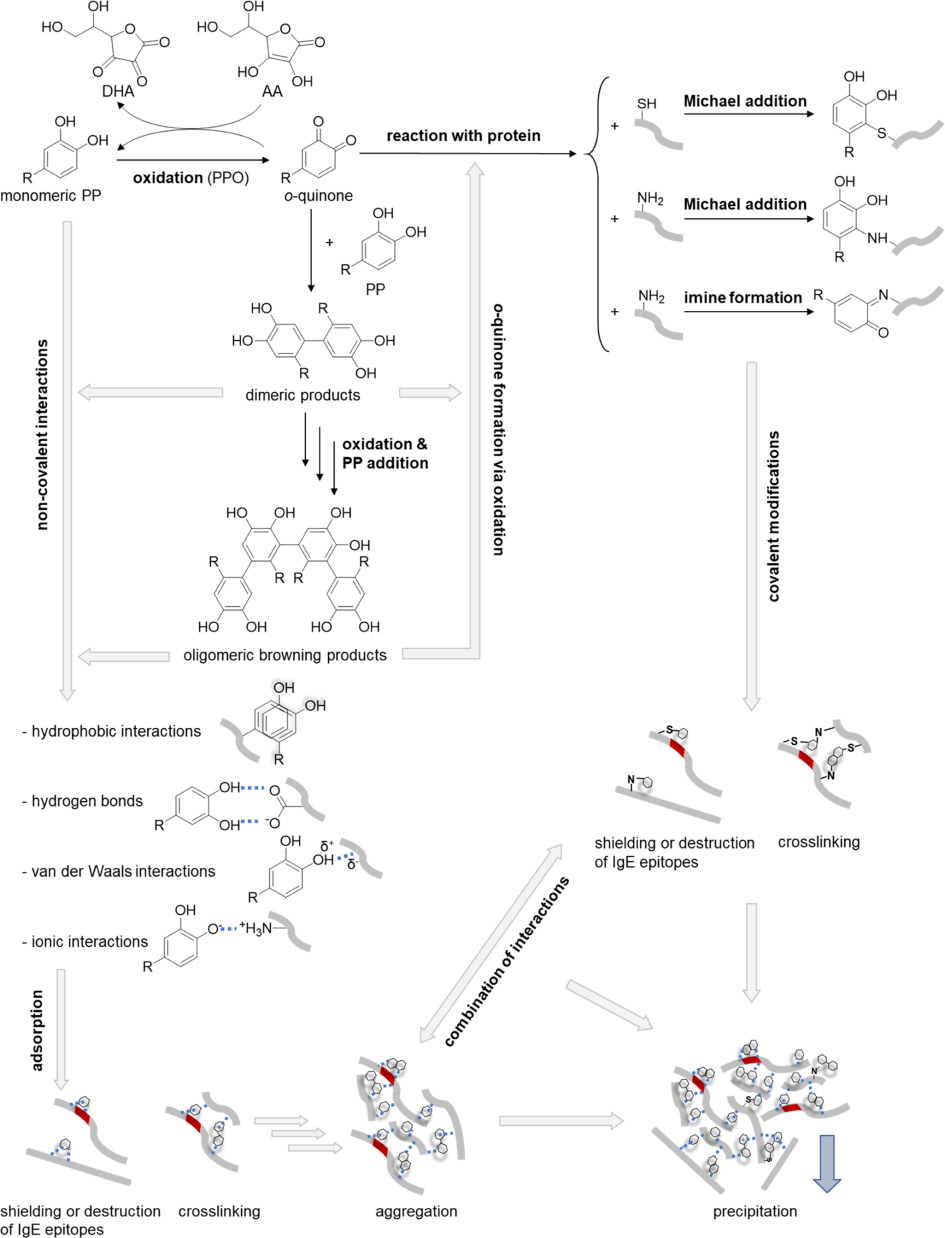
Overview of covalent bond formations (black lines) and noncovalent interactions (blue dotted lines) between polyphenols (PP, simplified as hexagons) and proteins (simplified as bold gray lines), which might lead to a reduced allergenic potential, due to shielding or destruction of the allergen epitope (red).^15,18−22^ AA, ascorbic acid; DHA, dehydroascorbic acid; and PPO, polyphenol oxidase.

Previous studies indicate that PP are able to modify the allergenic potential of different foods.^15,16^ It is well known that PP and amino acid side chains of proteins interact with each other, either by the formation of covalent bonds or noncovalent interactions (Figure 1).^16−18^ For the latter hydrogen bonds, hydrophobic interactions, van der Waals forces, and ionic interactions occur.^15,17,18^ However, these noncovalent interactions are weaker than covalent bonds.^16^ Covalent bonds are formed between side chains of nucleophilic amino acids (cysteine, lysine, serine, tryptophan, methionine, histidine, tyrosine, and proline) and *o*-quinones, the highly reactive phenolic oxidation products (Figure 1).^16,19,20^ Both mechanisms might lead to shielding or destruction of immunoglobulin E (IgE) epitopes of Mal d 1 by changes in the protein structure, aggregate formation, and precipitation, which might reduce the allergenic potential.^15,16,21^

The formation of *o*-quinones is enzymatically catalyzed by endogenous polyphenol oxidase (PPO) in apples.^22^*o*-Quinones are important intermediates in browning of the fruits, since they form oligomeric condensation products in secondary reactions (Figure 1). For the high-molecular-weight products, an increased affinity to proteins is reported, which might lead to aggregation and precipitation.^18^ Ascorbic acid and other reducing agents reverse the formation of *o*-quinones.^22^ Kschonsek et al. demonstrated that the IgE binding of Mal d 1 was reduced for browned apples compared to unoxidized fruits.^7^ Gruber et al. reported a reduced IgE binding ability of the recombinant expressed PR-10 allergen r-Pru av 1, in a model system containing PP and commercial tyrosinase.^23^ No effect was obvious in the absence of the enzyme. In freeze-dried Golden Delicious peel samples, Garcia et al. observed a time-dependent reduction of Mal d 1, quantified by enzyme-linked immunosorbent assay (ELISA).^24^ The decrease in the Mal d 1 content was accelerated in the presence of the exogenic catechin. The fastest decline was observed when mushroom tyrosinase was spiked in addition to catechin.

The results of Garcia et al., Kschonsek et al., and Gruber et al. indicate that in addition to the PP content, the activity of the endogenous PPO in the apple and thus the *o*-quinone formation rate is important.^7,23,24^ Furthermore, a direct oxidizing effect of PPO on the amino acid tyrosine, first forming the *o*-diphenol (cresolase activity) and later the *o*-quinone product (catecholase activity), is also discussed.^7,24^

Mass spectrometry (MS) and ^1^H–^15^N-heteronuclear single quantum coherence (^1^H–^15^N-HSQC) NMR experiments by Ahammer et al. and Unterhauser et al. demonstrated that the cysteine at position Cys107 in the isoallergen 1.01 is susceptible to react covalently with ascorbic acid and oxidized chlorogenic acid in vitro.^25,26^ The covalent binding of both ligands to Mal d 1 resulted only in shifts in the resonance positions of the amide backbone signals of amino acids in proximity to Cys107, indicating that the overall tertiary structure of Mal d 1 was not affected.

A previous study by our group did not identify any correlation between the content of unoxidized PP and the allergenicity of an apple variety, which was reported in a consumer survey by the BUND Lemgo.^5,11^ However, a pronounced impact of the Mal d 1 content and the isoallergen profile on the allergenic potential was observed. As the allergenic reaction is restricted to the mouth and throat area, the amount of bioaccessible and thus potentially allergenic Mal d 1 during the oral phase is of interest. Additionally, since PP and Mal d 1 are localized in different cell organelles which come only into contact with each other after cell decompartmentation, interactions of PP with the allergen Mal d 1 have to occur before or during the oral phase.^27,28^ Therefore, the proportion of released Mal d 1 during in vitro oral digestion according to the COST protocol^29^ was determined for various apple samples and a model system containing recombinant expressed Mal d 1.01 (r-Mal d 1.01). Additionally, the interactions between PP and Mal d 1 in the presence and absence of PPO (tyrosinase) were studied by targeted and untargeted mass spectrometry, isothermal titration calorimetry (ITC), saturation transfer difference (STD), and ^1^H–^15^N-HSQC NMR spectroscopy in model systems with r-Mal d 1.01. The various analytical approaches are required because they provide partly complementary information about the binding event.^18^

## 2 Material and Methods

### 2.1 Chemicals

All chemicals were of analytical grade. For MS, analytical solvents and formic acid were of MS grade. Acetonitrile, acetone, and methanol were bought from Fisher Scientific (Loughborough, UK). Ammonium bicarbonate and iodoacetamide were acquired from Sigma-Aldrich (Taufkirchen, Germany). Urea, thiourea, Tris-hydrochloride, dithiothreitol, potassium chloride, potassium dihydrogen phosphate, and calcium chloride were bought from Roth (Karlsruhe, Germany). Hydrochloric acid (concentrated) and sodium hydroxide were purchased from Grüssing (Filsum, Germany), while formic acid, sodium hydrogen carbonate, magnesium chloride hexahydrate, and ammonium carbonate were acquired from Merck (Darmstadt, Germany). Trypsin, (sequencing grade) modified from porcine pancreases was bought from Serva (Heidelberg, Germany). Ultrapure water (ELGA Pure Lab Flex, Veolia Waters, Celle, Germany) was used throughout the experiments.

### 2.2 Sample Material

Freeze-dried apple flesh of the varieties Jonagold, Santana, Golden Delicious, and Goldparmäne harvested in 2019 were used for the experiments.^30^ Freeze-drying of apples was performed according to the procedure published previously.^13^ Information regarding the polyphenol profiles of the utilized apple samples has already been published.^11,13^

Recombinant Mal d 1.0108 (r-Mal d 1.01, Q9SYW3) for model systems was expressed in *Escherichia coli*.^31^ The r-Mal d 1.01 was stored in 20 mM Tris-HCl buffer at pH 7.5. To perform ^1^H–^15^N-HSQC NMR experiments *E. coli* was grown in M9 minimal medium with isotopically marked ammonium chloride (^15^NH_4_Cl, Sigma-Aldrich) as the sole nitrogen source. r-Mal d 1.01 concentrations in stock solutions for experiments were determined via UV–vis spectroscopy (ε = 14 900 L/(mol cm), calculated by the Expasy ProtParam tool).

Chlorogenic acid (CA), (−)-epicatechin (EC), (+)-catechin (CAT), and quercetin-3-glucoside (Q-glc) were purchased from Sigma-Aldrich (Taufkirchen, Germany), while procyanidins (PC) PC B1, PC B2 and PC C1 were acquired from PhytoLab (Vestenbergsgreuth, Germany). Oxidized EC (EC_ox_) was prepared by stirring a defined volume of 7.4 mM EC solution in sodium phosphate buffer (pH 7) for 42 h on air. Subsequently, the orange/brown solution was filled up to its previous volume with water to compensate for evaporation. The formation of high molecular oxidation products of EC was proven by the color change; however, no further structure elucidation of the product mixture was performed. The concentrations used in experiments are based on the originally prepared monomeric EC concentration. Concentrations of the PP stock solutions were determined by UV–vis spectroscopy (Spectrostar Nano, BMG Labtech, Ortenberg, Germany) with absorption coefficients published previously.^32^

### 2.3 Release of Mal d 1 from Apple Flesh

To quantify the Mal d 1 release with simulated saliva fluid (SSF) 1 g of apple flesh of the hypoallergenic variety Santana and the two commercial varieties Golden Delicious and Jonagold, all characterized by a low total phenolic content, and the traditional and polyphenol-rich variety Goldparmäne were rehydrated with 5.21 mL of water. Then, 1 mL of SSF (KCl = 15.1 mmol/L, KH_2_PO_4_ = 3.7 mmol/L, NaHCO_3_ = 13.6 mmol/L, MgCl_2_(H_2_O)_6_ = 0.15 mmol/L, (NH_4_)_2_CO_3_ = 0.06 mmol/L, CaCl_2_ = 1.5 mmol/L, adjusted with HCl to pH 7 and without α-amylase since apples are low in starch and no effect on Mal d 1 release is expected) was added according to the COST protocol for in vitro digestion.^29,33^ The samples were mixed at room temperature (RT) for 45 s and then immediately centrifuged and analyzed by targeted mass spectrometry.

In vitro oral digestion experiments were performed according to the COST protocol.^29^ Samples of the varieties Goldparmäne and Golden Delicious were incubated before centrifugation in an overhead shaker for 2 min at 37 °C. In order to study the effect of the temperature, in particular as the temperature of an apple bite is different from 37 °C at the beginning of the oral phase, the samples were additionally incubated for 2 min at RT.

To test for a possible loss of endogenous PPO activity due to freeze-drying, some samples were supplemented with 1.7 μg of dissolved mushroom tyrosinase from *Basidiomycota* (activity: 8503 units/mg, Sigma-Aldrich, St. Louis, MO). However, no effect of the PPO was observed and therefore, the different samples were treated as replicates in data analysis.

### 2.4 Simulation of Enzymatic Browning

Samples of Goldparmäne, Golden Delicious, Santana, and Jonagold were rehydrated and oxidized in the presence of air for 20 min at RT. Subsequent to incubation/oxidation samples were tryptically digested and analyzed as described previously.

### 2.5 Spiking Experiments with Epicatechin (EC) and Chlorogenic Acid (CA)

To study the effect of PP on the extractability and bioaccessibility of Mal d 1 during oral digestion Golden Delicious, characterized by its rather low phenolic content in the flesh with only 0.5 mg/g CA and 0.2 mg/g EC dry weight (DW), was used.^11^ The amount of 2 mg CA and 1 mg EC, dissolved in water, were spiked to the samples, representing approximately the amounts of these phenolics in polyphenol-rich apples (e.g., Bohnapfel with 1.7 mg/g DW (CA) and 0.9 mg/g DW (EC)).^11^ In addition, to better understand the effect of elevated phenolic contents, samples with double the amounts of these compounds were prepared.

### 2.6 Targeted Mass Spectrometry of Apple Samples

After experiments, samples were centrifuged at 4 °C and 10 104  rcf for 30 s. The proteins in 250 μL of the supernatant were precipitated with 1.5 mL of ice-cold acetone and isotopically labeled standard peptides (Spike Tides TQL, JPT, Berlin, Germany) (Figure S1) were added. The samples were tryptically digested and quantified by targeted MS as described previously.^34^ Data analysis was performed with Skyline (version 22.2.0.527, MacCoss Lab, Department of Genome Sciences, UW).

### 2.7 Targeted and Untargeted Bottom-Up Mass Spectrometry of r-Mal d 1.01 Model Systems

Untargeted MS allows for qualitative detection of modified peptides, which is challenging with targeted MS where only precursors with a previously specified *m*/*z* are fragmented. However, targeted MS experiments are required to quantify the specified marker peptides, thus enabling the determination of the extent to which marker peptides have been affected by modifications.

For targeted MS experiments 50 μL of r-Mal d 1.01 (β = 0.8 mg/mL) were mixed with 150 μL of SSF containing CA and EC. The mass ratio of r-Mal 1.01 to CA and EC was 1:10 and 1:5, respectively. The ratio of r-Mal d 1.01 to CA and EC in the model system approximates the ratio found in Bohnapfel.^11,30^ In addition, model systems with 1.4 μg/mL mushroom tyrosinase were prepared. After incubation of the samples for 20 min at RT, 125 μL of the solution was mixed with 125 μL of protein extract from Santana flesh to guaranty^34^ a reproducible tryptic digestion. The amount of Mal d 1.01 contributed by the Santana flesh was 3 μg, in addition to the 25 μg of r-Mal d 1.01 in each sample.

For untargeted MS analyses samples with r-Mal d 1.01 (β = 1 mg/mL, 57 μM), EC (β = 4 mg/mL, 13.7 mM), or mushroom tyrosinase (β = 0.01 mg/mL) or both were prepared in water, with a final volume of 100 μL. The molar ratio of r-Mal d 1.01 to EC was 1:240 and corresponds to the ratio used in the spiking experiment with Golden Delicious. Samples were incubated at RT and 6 M urea buffer was added after 20 min, followed by tryptic digestion without the addition of isotopically labeled standards. As control, r-Mal d 1.01 was digested immediately without the addition of PP or mushroom tyrosinase. Samples were analyzed via untargeted high-resolution mass spectrometry as described previously.^31^ Data was processed by PeaksX (Bioinformatic Solution Inc., Waterloo, Canada) to identify r-Mal d 1.01 covalently bound to EC by a database-assisted search. According to Börsig et al.,^19^ the following settings were used: semispecific digestion mode for trypsin with a maximum of five missed cleavages and five post-translational modifications per peptide, a parent mass error tolerance of 50 ppm and fragment mass error tolerance of 0.05 Da. The expected mass shifts according to the Michael addition in the modified peptides were included for monomeric (mEC, Δ*m* = 288.06 DA), dimeric (dEC, Δ*m* = 576.13 DA), and trimeric (tEC, Δ*m* = 864.19 DA) EC as customized post-translational modifications (PTMs). Permitted modification sites were restricted to the amino acids serine, cysteine, lysine tyrosine, and phenylalanine. The basis for the database search was the amino acid sequence of isoform Mal d 1.0108 (Q9SYW3).

### 2.8 Isothermal Titration Calorimetry (ITC)

Experiments were conducted on a Nano ITC (TA Instruments, Eschborn, Germany), in 0.1 M sodium phosphate buffer, pH 7. Previous to the experiments, all solutions were degassed at 277 K (4 °C) for 15 min. Experiments were conducted at 298 K (25 °C) with a stirring rate of 125 rpm, a start delay of 200 s, and a medium peak height-to-width ratio. For experiments, 320 μL of 0.2 mM r-Mal d 1.01 solution was added to the sample cell of the ITC, and 50 injections of 2 μL of 4 mM phenolic stock solutions (PC B1, PC B2, PC C1, EC, CAT, and EC_ox_) were added to the protein solution with a time interval of 250 s. Samples were analyzed in duplicate. Data analysis was performed with NanoAnalyze Software (TA Instruments, Eschborn, Germany). Data was corrected for non-reaction-related heats by applying the average of the three lowest heat values at the end of the experiment as constant blank. The data was fitted by “independent” mode. The stoichiometry *n* was fixed at 1, leaving only the association constant *K*_a_ and the change in enthalpy (Δ*H*) as variables. Setting the stoichiometry is required for low-affinity systems to prevent overparameterization during curve fitting.^35^ Blank measurements confirmed that heat generation due to the dilution of the injected PP solution was neglectable.

### 2.9 ^1^H–^15^N-Heteronuclear Single Quantum Coherence (HSQC) and Saturation Transfer Difference (STD) NMR Experiments

Data was acquired on a Bruker Advance III HD 700 NMR spectrometer equipped with a 5 mm QCI cryo probe head with ATM and z-gradient and BCU I cryostat for sample temperature control. Spectra were recorded at 298 K (25 °C). ^1^H–^15^N-HSQC NMR experiments were carried out to determine potential binding sites of various PP on Mal d 1 and to monitor the conformational integrity of the allergen. Samples with 100 μM isotopically labeled r-Mal d 1.01 and 0.1, 1 and 2 mM EC, EC_ox_, CA, and PC C1 were prepared in 50 mM phosphate buffer pH 7, containing 10% D_2_O. Due to its low solubility at aqueous conditions, the concentrations of Q-glc were reduced to 0.05, 0.1, and 0.15 mM. To examine the impact of PPO on Mal d 1, mushroom tyrosinase was added to one sample at a molar ratio of 1:20 (mushroom tyrosinase: r-Mal d 1.01). For ^1^H–^15^N-HSQC NMR experiments the pulse program hsqcetfpf3gpsi with a spectral width of 16 ppm for ^1^H and 80 ppm for ^15^N was employed. All two-dimensional ^1^H–^15^N-HSQC NMR spectra were recorded with 4096 data points in F2 and 512 increments using nonuniform sampling (NUS = 25%). In total, 32 scans/increments were acquired, and the recycle delay D1 was set to 2 s. To test for protein integrity and reduction of signal intensity due to precipitation, a significantly shorter 1D ^1^H–^15^N-HSQC NMR experiment was performed. For this, 128 scans with an acquisition time of 0.73 s and a spectral width of 16 ppm were collected. Data was analyzed by TopSpin (Version 4.0.6, Bruker). Signal assignment in the ^1^H–^15^N-HSQC NMR spectra is based on the data published by Ahammer et al.^36^

To determine the epitope of the phenolics on Mal d 1, STD-NMR experiments were carried out with 10 μM r-Mal d 1.01 and 1 mM PP in 100 mM phosphate buffer (90% D_2_O/10% H_2_O) at 298 K (25 °C). For the experiment, a standard Bruker pulse sequence (stddiffgp19.3) was used, in which the HDO resonance is suppressed through a watergate sequence. Selective saturation of the protein was achieved by a train of Gauss-shaped pulses of 50 ms length each, truncated at 1%, and separated by a 1 ms delay. Forty selective pulses were applied, leading to a total length of the saturation train of 2 s. The on-resonance irradiation of the protein was performed at a chemical shift of 0.5 ppm. Off-resonance irradiation was set at 100 ppm, where no protein signals are present. Thus, a protein saturation of 60% was achieved.

## 3 Results

### 3.1 Release of Mal d 1 from Apple Flesh

**Figure 2:**
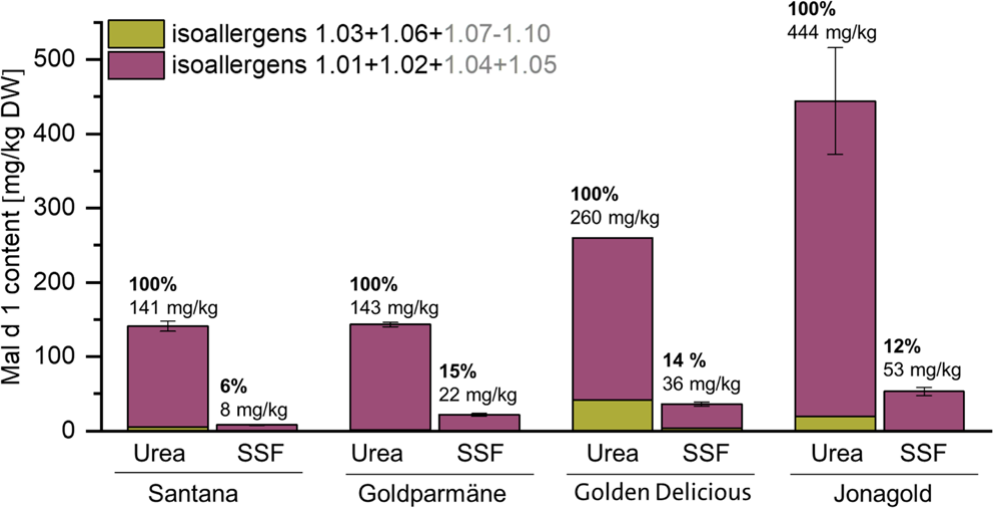
Quantified Mal d 1 content and proportion of release in samples extracted with urea buffer and simulated saliva fluid (SSF) at the beginning of the oral phase.

Only 6–15% of the total Mal d 1 content (comprehensive extraction with urea) in the apple flesh was extracted by SSF (quantified by the global marker IAPQAVK and IAPQAIK, Figure 2). A similar trend was observed, quantifying the isoallergen-specific markers, even if the sum of these was lower than the total Mal d 1 content quantified using global markers (Figure S2). These results indicated that a markedly reduced proportion of the total Mal d 1 causes allergic symptoms. Despite significantly different initial Mal d 1 contents, similar proportions were released from Jonagold, Goldparmäne, and Golden Delicious. This indicated an analogous matrix effect independent of the variety. For Santana, a significantly lower release of Mal d 1 was observed during the oral phase, which might explain the better tolerability often reported for this variety.^37^ However, the high degree of milling in comparison to that of the apple bolus after chewing might affect the results. For raw carrots, a particle size of approximately 2.0–6.5 mm is reported for 60% of the total masticated mass after chewing.^38^ A similar distribution of particle sizes might be assumed for raw apples. It is therefore likely that the quantified content of released Mal d 1 in the in vitro oral digestion experiments was overestimated, and ex vivo experiments with freshly consumed apples are recommended.

The quantified proportion of released Mal d 1 in in vitro experiments without mushroom tyrosinase (data not shown) indicates that the addition of tyrosinase did not impact the release of Mal d 1 during in vitro digestion and browning. Due to the high acidity of the apple flesh and the low buffering capacity of SSF, the pH of the samples was around ∼3.5. At this pH, the enzyme activity of mushroom tyrosinase is probably significantly reduced because an optimum at pH 7 and a loss of activity at pH 4.5 is reported.^39^ For endogenous apple PPO, pH optima of 4–5 have been determined in previous experiments (data not shown).

### 3.2 Effect of Phenolics on Mal d 1 Release from Apple Flesh

According to the COST protocol the oral phase is estimated to last for 2 min, in which the food is crushed.^29^ Since PP, Mal d 1, and endogenous PPO are separated in an intact apple cell, they come only into contact after cell decompartmentation.^27,28^ Thus, the formation of covalent bonds and noncovalent interactions, affecting the allergenic potential of Mal d 1, is restricted to this time frame.

**Figure 3:**
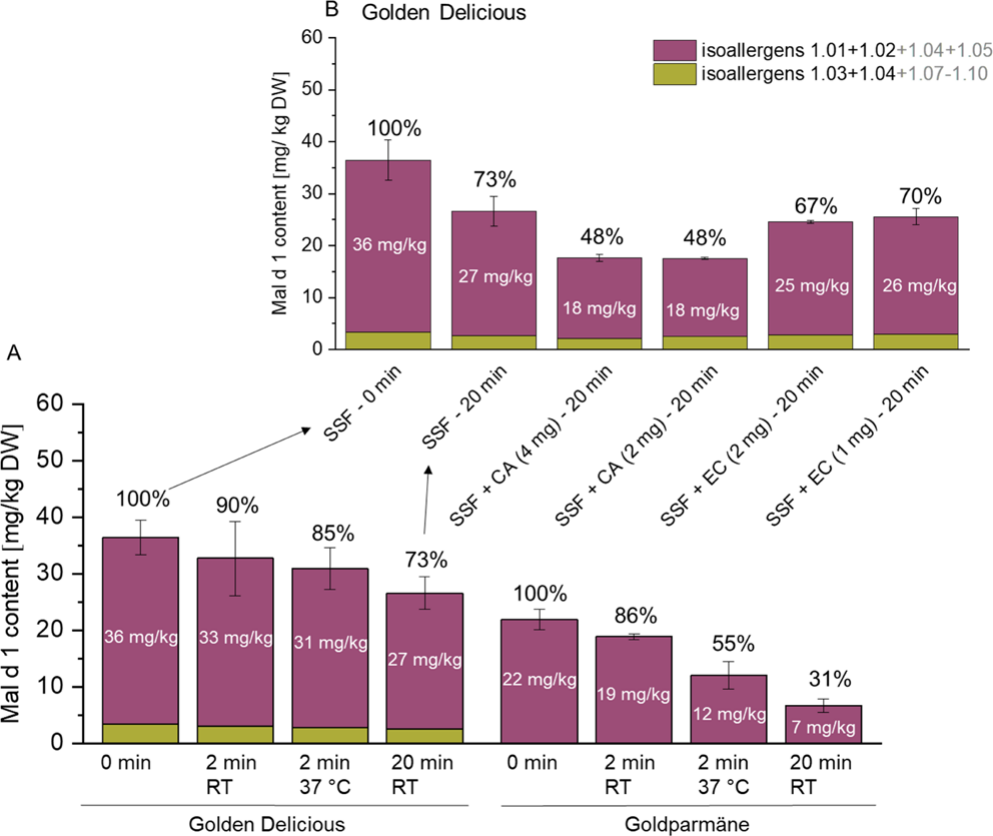
(A) Mal d 1 quantified under different in vitro oral digestion conditions in flesh from Golden Delicious and Goldparmäne. (B) Effect of supplementation with epicatechin (EC) and chlorogenic acid (CA) in Golden Delicious flesh after incubation for 20 min at room temperature. Mal d 1 quantified is given in white numbers, and the proportion (black) is based on the initial (*t* = 0) release of Mal d 1 in SSF.

Our results for the simulated oral digestion of apple flesh show a time-dependent decrease in the quantified Mal d 1 content (Figure 3A, isoallergen-specific markers, Figure S3). The decline was amplified at 37 °C and more pronounced in Goldparmäne, a variety with a high phenolic content,^13^ than in Golden Delicious, a PP-deprived variety.^11^ To simulate browning on air, samples were incubated for 20 min at RT. In Goldparmäne, only 31% of the initial Mal d 1 content was quantified. In contrast, in Golden Delicious 73% of the initial allergen content was released. A similar trend was observed for the varieties Santana (53%) and Jonagold (87%), both are of commercial importance and characterized by low phenolic contents.^13,11^

Supplementation with exogenous CA and EC to Golden Delicious samples and incubation for 20 min confirmed that the amount of quantified Mal d 1 decreased with increasing phenolic contents (Figure 3B). Adding CA, the Mal d 1 content decreased from 73 to 48%, while this effect was less pronounced for EC (70–67%). This indicates, an interaction of Mal d 1 in particular with CA, which is one of the main PP in apples. It was expected that a higher concentration of CA or EC would result in a further decreased Mal d 1 content; however, no correlation was observed between the amount of PP added and the Mal d 1 quantified (Figure 3B). This might be due to saturation of the endogenous PPO in the samples.

The subsequent decline in the Mal d 1 content quantified with increasing incubation time indicates that the oxidation products of the PP (browning products) and not the endogenous monomeric PP were the cause for the decrease in the soluble allergen content (Figure 3A). Oxidation of PP is catalyzed by the PPO, and therefore, dependent on the turnover rate. Previously, it has been demonstrated that browning products might inhibit the enzyme at elevated concentrations.^40^

### 3.3 Effect of Epicatechin (EC) and Chlorogenic Acid (CA) on r-Mal d 1.01 in a Model System Containing Mushroom Tyrosinase

**Figure 4:**
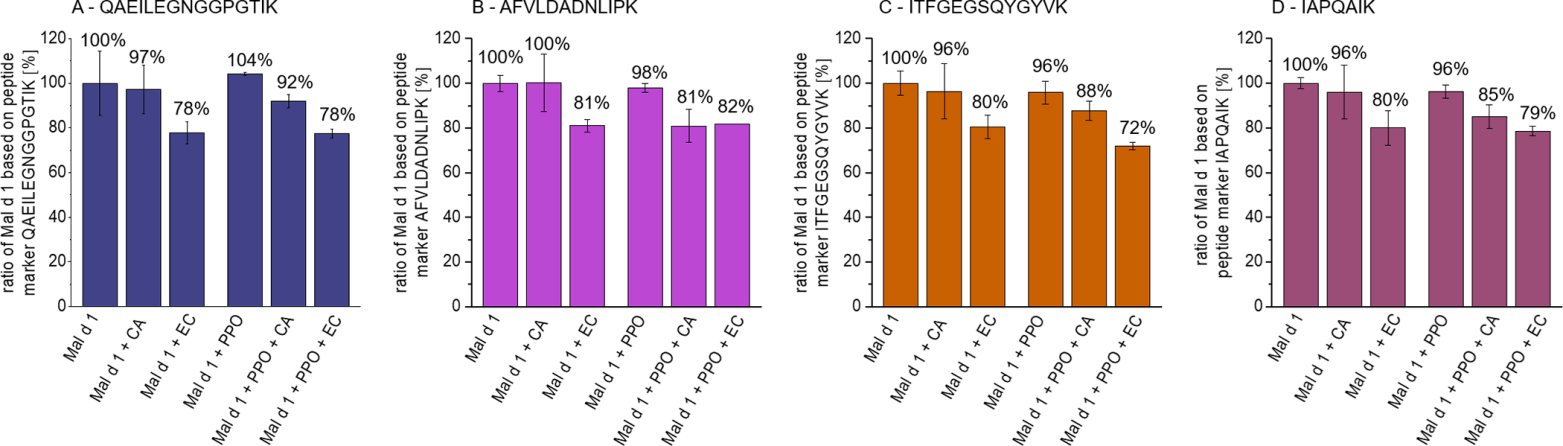
Effect of chlorogenic acid (CA, 0.4 mM), epicatechin (EC, 0.2 mM), and exogenous mushroom tyrosinase (PPO) on four different marker peptides quantified in a model system with r-Mal d 1.01. All samples were incubated for 20 min at room temperature. Proportions were calculated based on the quantified marker content in the model system without PP and mushroom tyrosinase.

Since r-Mal d 1.01 was the only isoallergen in the model system, the respective markers QAEILEGNGGPGTIK, AFVLDADNLIPK, ITFGEGSQYGYVK, and IAPQAIK were quantified to evaluate specific interaction sites (Figures 4 and S1). However, the decline during incubation with and without additional EC, CA, and mushroom tyrosinase was analogous for these markers. This suggests that there is not a specific interaction site, at least in the region covered by the four markers (amino acids 22–69). Most probably the interaction with PP impacts the whole protein, e.g., by the formation of nonsoluble PP-Mal d 1 and/or nondigestible complexes. Furthermore, the marker ITFGEGSQYGYVK (Figure 4C) was not affected by mushroom tyrosinase in the absence of PP, indicating no direct oxidization of the tyrosine by the enzyme.

In contrast to the apple flesh samples, the decrease in r-Mal d 1.01 was more pronounced in the presence of EC than CA. While for EC this effect was independent of the presence of mushroom tyrosinase, CA reduced the quantified allergen content only if the enzyme was present. This observation might be explained by the different pH values of the model systems. The neutral pH of the model system with EC permitted autoxidation, formation of *o-*quinones without the aid of PPO, of the PP. Due to the p*K*_A_ value of CA the pH of the model system dropped to 4.5. The slower autoxidation of PP under acidic conditions, in particular of CA than EC, has already been reported in the literature.^41^

### 3.4 Isothermal Titration Calorimetry (ITC) Experiments to Determine Binding Constants between Mal d 1 and Polyphenols

**Figure 5:**
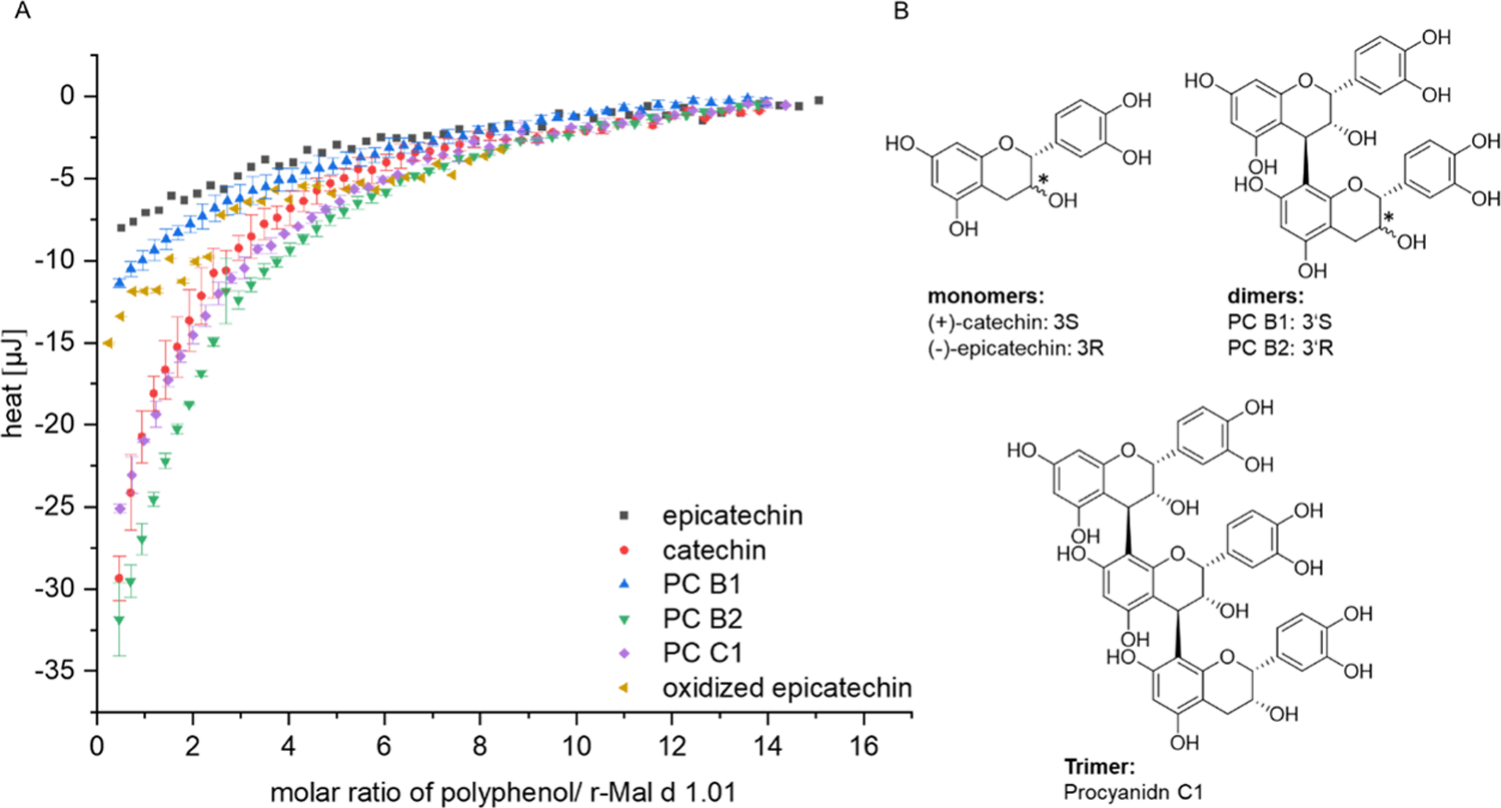
(A) Wiseman plot for the interaction of r-Mal d 1.01 with flavanols. (B) Structures of the studied flavanols. Differences in the stereo centers of monomers and dimers are marked by an asterisk. PC, procyanidin.

Noncovalent interactions between r-Mal d 1.01 and PP tested were generally characterized by a low heat release and high *K*_D_ values in the mM range (Figure 5, Table S4). This suggests that neither the formation of covalent bonds nor specific interactions in a binding pocket are involved, but rather nonspecific adsorption on the protein surface. Differences were observed between EC and CAT and to a lesser extent between PC B1 (one catechin and one epicatechin monomer) and PC B2 (two epicatechin monomers; Figure 5, Table S4). These structures only vary in one stereocenter, and therefore, it must be assumed that the binding affinity of flavanols to Mal d 1 is significantly affected by their steric properties.

Due to the low affinity, it was required to preset the stoichiometry to prevent overparameterization of the fit of the Wiseman plot.^35^ Therefore, a stoichiometry of *n* = 1 was chosen, which limits data interpretation, and the thermodynamic values provided in the Supporting Information must be considered with caution (Table S4). In general, data for oxidized EC must be interpreted tentatively, as the concentration is related to the monomeric EC in the unoxidized stock solution.

All interactions observed were exothermic, which is in general associated with van der Waals forces, hydrogen bonds, and electrostatic interactions.^42^ Positive changes of entropy (Δ*S* positive) are an indicator for the release of water molecules from the solvation “cages” of hydrophobic groups (binding pocket), while negative changes signify a loss of freedom in the system or conformational changes.^42,43^ Therefore, our results indicated weak adsorption with no specific binding epitope of the PP on the r-Mal d 1.01 surface. The slightly higher impact of Δ*H* than *T*Δ*S* on Δ*G* might indicate an interaction based on hydrogen bonds and van der Waals forces.^42^ The positive Δ*S* values suggest additional hydrophobic interactions, but it must be kept in mind that uncertainties in Δ*H* result in an opposite error in *T*Δ*S* of the same magnitude.^42,44^

### 3.5 Covalent Bond Formation between Polyphenols and r-Mal d 1.01 Identified by Bottom-Up Untargeted Mass Spectrometry

**Figure 6:**
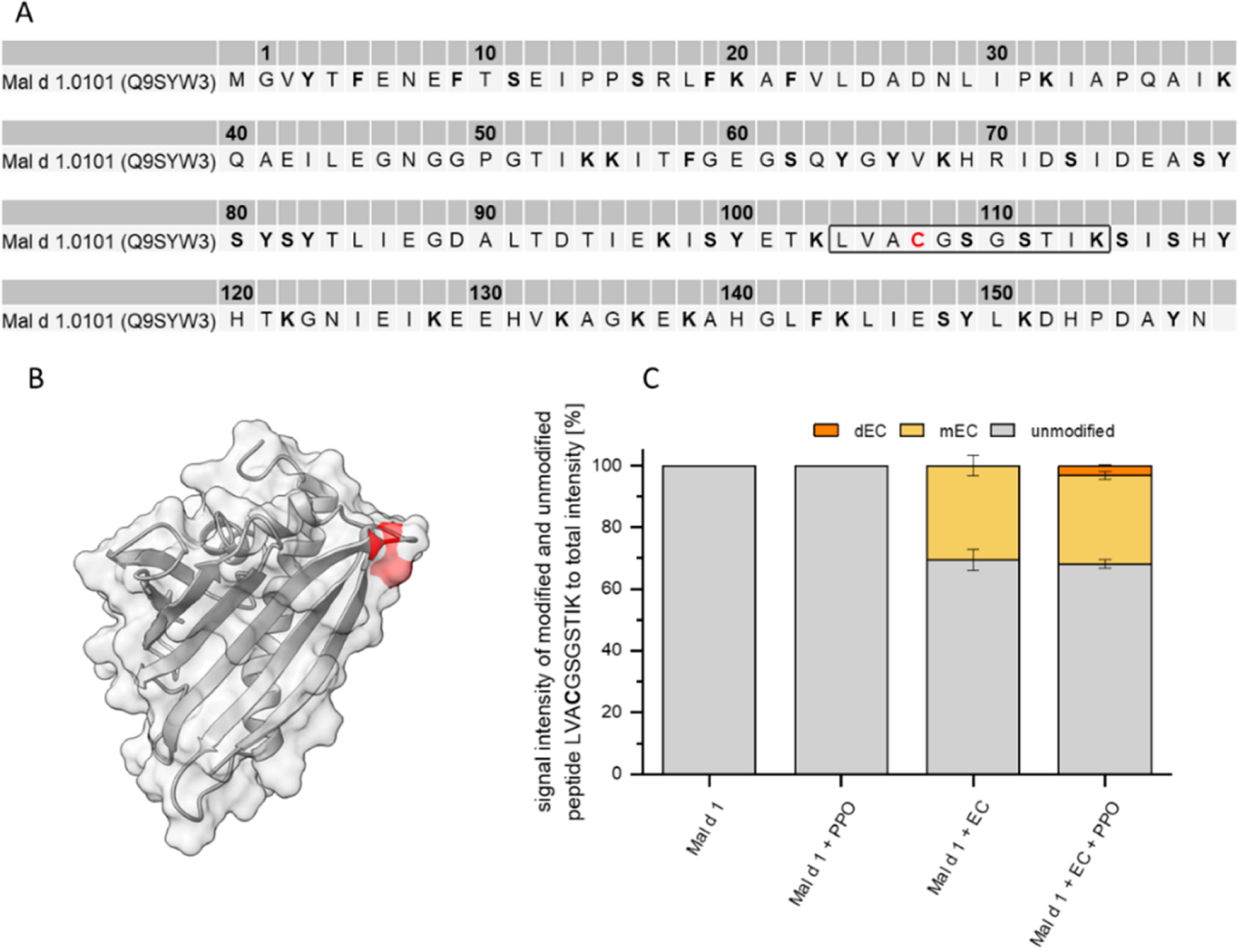
Modifications of r-Mal d 1.01 (100 μM) in the presence of epicatechin (EC, 400 μM) with and without mushroom tyrosinase (PPO). (A) Amino acid sequence of r-Mal d 1.01. Modification sites, included in the search algorithm, are highlighted in bold. The peptide LVACGSGSTIK contains the modified cysteine Cys107. (B) Position of cysteine Cys107 (red) in r-Mal d 1.01. (C) Proportion of the signal intensity of the modified peptide LVACGSGSTIK to the total intensity of the peptide. mEC = modification by a monomeric epicatechin, dEC = modification by a dimer of epicatechin.

The incubation of r-Mal d 1.01 with an excess of EC resulted in the modification of the cysteine Cys107 (peptide LVACGSGSTIK, Figure 6A,B) by Michael addition. Covalent interactions by imine formation were not detected, since only the Δ*m* values for EC bound via the Michael addition were included as post-translation modifications in the search parameters. The proportion of the signal intensities indicated a modification of approximately 30% by EC-monomer (Figure 6C). For EC, the *o*-quinones, required for covalent bond formation, are formed at neutral pH without the aid of PPO.^41^ In the presence of mushroom tyrosinase, also modifications by EC-dimers were detected (3%). When interpreting the ratio of the signal intensities of modified and unmodified r-Mal d 1.01, it is important to note that the tryptic digestibility of modified and unmodified protein and the ionization capability during mass spectrometry are not necessarily comparable. Analysis of samples after incubation with and without mushroom tyrosinase and EC addition by SDS-PAGE did not confirm Mal d 1 cross-linking although insoluble protein precipitates had formed after 3 days.

In contrast to the model systems, no covalent modifications of Mal d 1 with EC or CA were detected in apple samples (Golden Delicious and Goldparmäne incubated for 20 min at RT), independently of mushroom tyrosinase addition (data not shown). This might suggest either that the reaction of cysteine with polyphenols does not occur in apples or, most probably, that the modifications were not detected due to their low abundance. In the applied data-dependent acquisition mode, only the most intense peptides in the apple samples were fragmented.

### 3.6 Saturation Transfer Difference (STD) NMR Experiments Failed to Determine Interaction Sites

Only 3% of saturation was transferred from r-Mal d 1.01 to PC B2 (Figure S5B), which was close to the ligand saturation of 2% in the absence of the protein (Figure S5A). This might indicate that the PC B2 does not interact with the allergen or it could be a result of the less effective spin diffusion (60% after pulse optimization), related to the low molecular weight of Mal d 1.

### 3.7 ^1^H–^15^N-Heteronuclear Single Quantum Coherence NMR Experiments Identified Amino Acids Affected by Polyphenol–Mal d 1 Interactions

**Figure 7:**
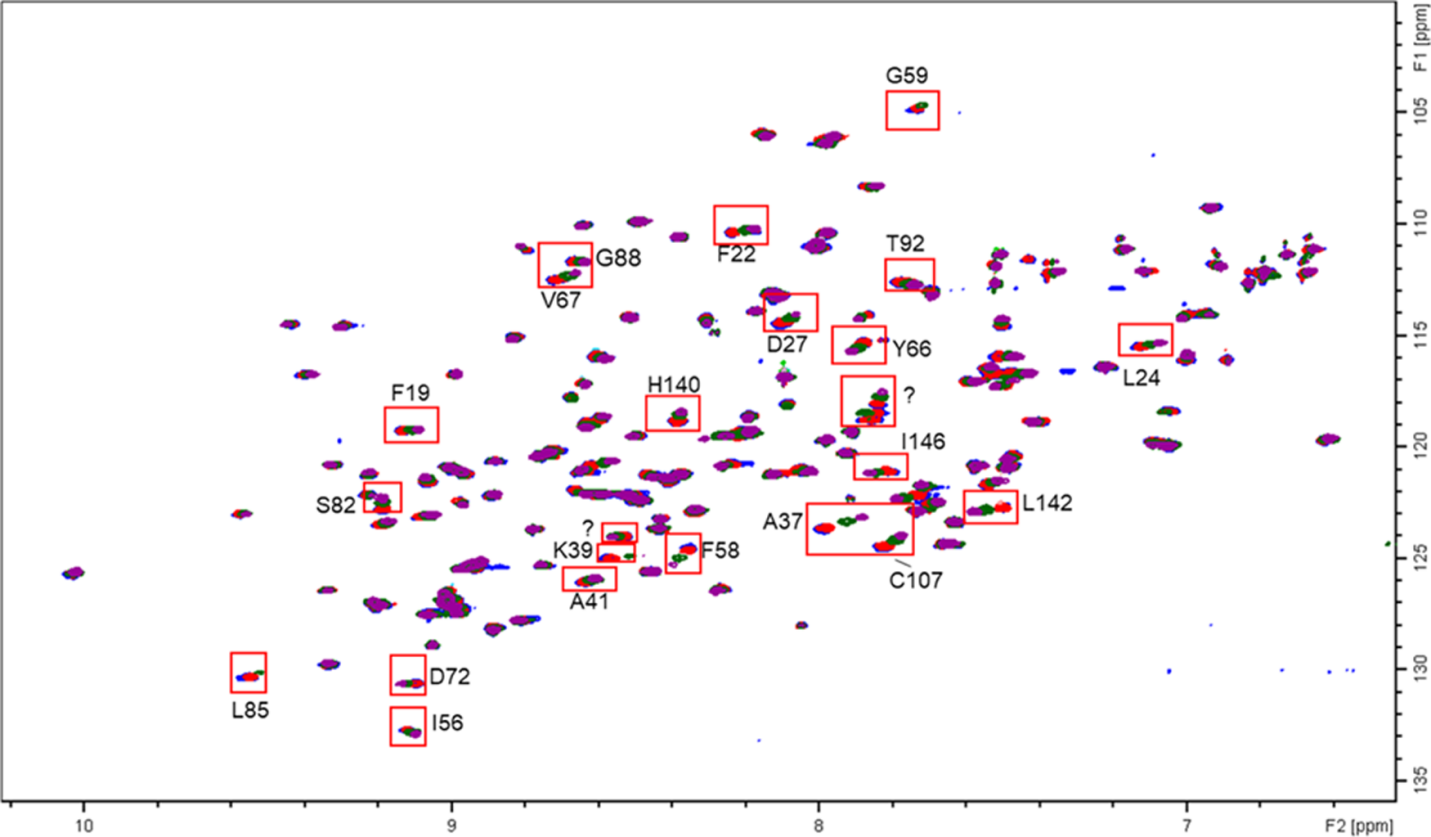
Overlay of ^1^H–^15^N-HSQC NMR spectra of the backbone amide protons of 100 μM uniform labeled r-Mal d 1.01 (blue) in the presence of 0.1 mM (red), 1 mM (green), and 2 mM (violet) chlorogenic acid. Assignment is provided for signals which undergo significant chemical shift perturbations. A question mark indicates that assignment to a specific amino acid was impossible.

The ^1^H–^15^N-HSQC NMR spectrum of r-Mal d 1.01 were similar to the data published by Ahammer et al., which allowed a straightforward signal assignment of the majority of amino acids in r-Mal d 1.01.^36^ Addition of different CA (Figure 7), EC (Figure S6A) and Q-glc (Figure S6B) concentrations to r-Mal d 1.01, resulted only in low chemical shift perturbations compared to free r-Mal d 1.01. This indicates that monomeric PP did not induce conformational changes. This observation was further supported by circular dichroism measurements, where no differences were observed in the r-Mal d 1.01 spectra in the presence and absence of EC and PC B2 (data not shown).

**Figure 8:**
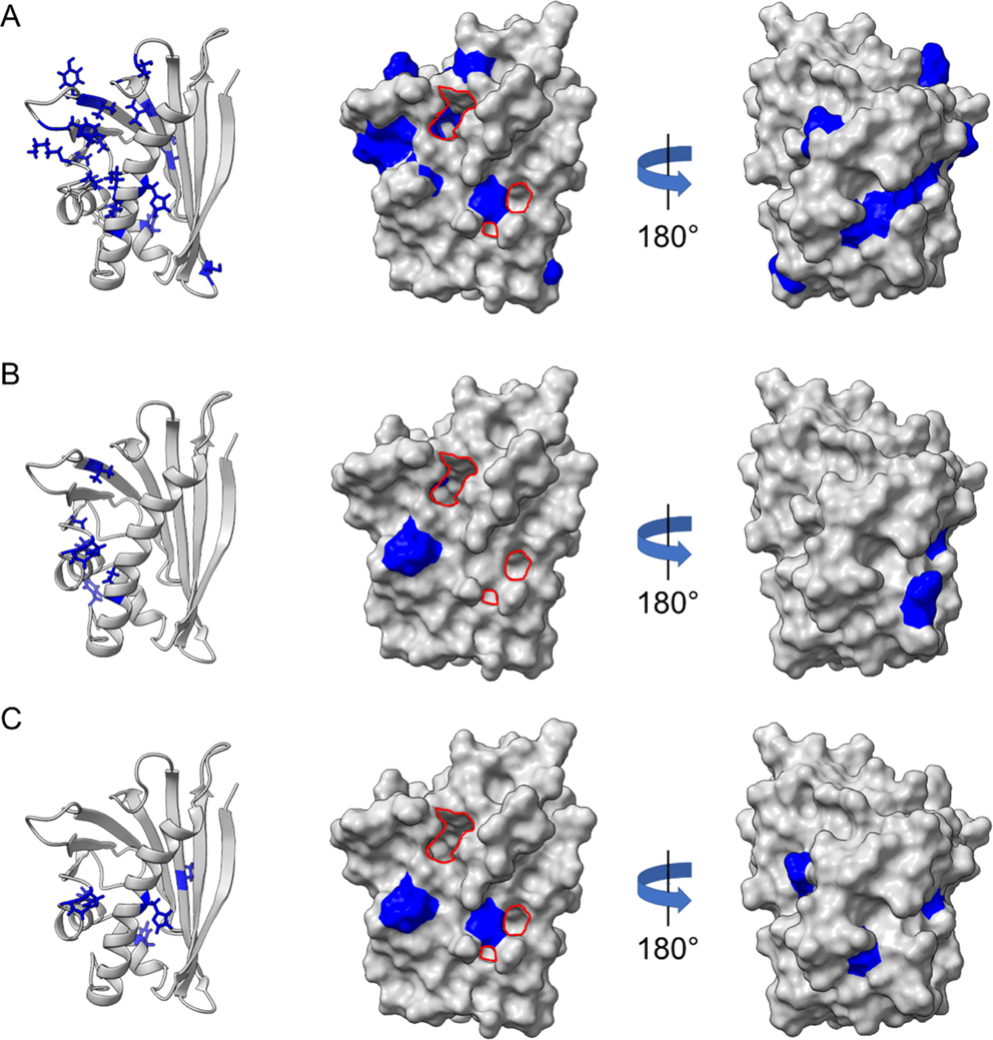
Position of amino acids and their side chains affected by concentration-dependent shifts in the amide backbone signal in ^1^H–^15^N-HSQC NMR (blue) in the presence of chlorogenic acid (A), epicatechin (B), and quercetin-3-glucoside (C) as ribbon diagram and surface model. Entrances to the internal cavity of Mal d 1 are marked in red. Visualization by UCSF ChimeraX (version 1.6.1), based on conformation data by Ahammer et al.^2^

The number and amino acid positions being affected differed between the monomeric PP studied (Figure 8). It is apparent that CA showed by far the strongest impact on the protein surface with an effect on 23 amino acids, followed by EC (10) and Q-glc ((7); Figures 7 and S6). Exclusively, aspartic acid at position 27 and alanine at position 37 were affected by all phenolics in a concentration-dependent manner. CA signals often shifted for amino acids in the direct vicinity of each other, indicating that larger areas of the allergen were influenced (Figure 8A). In contrast, in samples containing Q-glc and EC the affected amino acids were isolated from each other (Figure 8B,C). The low number of amino acids affected and the small shifts in the signals of the backbone amides by Q-glc might be explained by its significantly reduced concentrations. Since the tested concentrations of EC and CA were the same, our data indicated that EC interacts with fewer amino acids of r-Mal d 1.01 and to a lesser extent than CA. In general, each PP affected different amino acids; however, those were constant with increasing concentrations. Thus, there is not a specific binding site of r-Mal d 1.01 and interactions are scattered over the protein surface.

**Figure 9:**
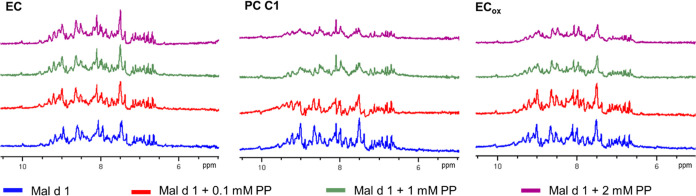
Effect of increasing concentration of polyphenols (PP) on the amide proton signal intensity of 100 μM r-Mal d 1.01 in the partial 1D-^1^H^15^N-HSQC NMR spectra.

While signal intensities of the r-Mal d 1.01 amide protons interacting with monomeric PP were stable, independent of the PP concentration used, a decline was observed in the system containing trimeric PC C1 and oxidized epicatechin. This effect is best illustrated by the 1D-projection of the ^1^H–^15^N-HSQC NMR, representing the signals of all amide protons (Figures 9 and S7). The reduced signal intensity suggested protein aggregation, which was confirmed by precipitation in those samples 24 h after spectra acquisition. These observations underline that oligomeric PP induce aggregation of r-Mal d 1.01 in an aqueous buffer.

The addition of mushroom tyrosinase did not affect the ^1^H–^15^N-HSQC NMR spectra (Figure S8), indicating that cross-linking of Mal d 1 via oxidized tyrosine side chains can be excluded.

## 4 Discussion

### 4.1 Release of Mal d 1 and Its Stability during In Vitro Oral Digestion and Enzymatic Browning

Since Mal d 1 is denatured during gastric digestion, it is of particular interest to estimate the soluble allergen content that triggers an immune response during the oral phase. The in vitro digestion experiments demonstrated that only a small proportion of the total Mal d 1 is released from the apple matrix (6–15%, Figure 2). The rate was similar among the varieties Golden Delicious, Goldparmäne, and Jonagold, while Santana, a variety advertised for its good tolerability,^5,37^ was characterized by an even further reduced release. This suggests that in addition to variances in the total Mal d 1 contents and profile, matrix-release characteristics during consumption might have an impact on the variety-specific allergenicity.

In addition to the Mal d 1 content and profile^9,11,30^ it is discussed that the allergenicity of an apple variety is affected by the polyphenols^3,7,14^ present in the fruit. It is assumed that phenolic structures interact with the allergen through covalent bond formation or by noncovalent interactions, both shielding IgE epitopes or aggregating the allergen (Figure 1).^15^ According to the COST protocol, the oral phase lasts for 2 min,^29^ although this is probably an overestimation of the dwell time of apples in the oral digestion phase. For example, Watanabe et al. determined a chewing time of approximately 30 s for 10 g apple.^45^ In this brief time frame, cell decompartmentation occurs enabling PP, Mal d 1, and PPO to come into contact.

The lowest levels of Mal d 1 were quantified in apple samples incubated for 20 min, indicating a significant impact of time on the soluble allergen content (Figure 3A). Prolonged incubation times allowed for the formation of browning products, which seem to be more potent in reducing the free Mal d 1. The more pronounced decrease in the quantified Mal d 1 content in Goldparmäne (PP-rich variety) compared to Golden Delicious (variety with a low PP content) after 20 min incubation indicated that the amount of PP affects the soluble and thus allergenic Mal d 1 content. This observation was further confirmed by the spiking experiments, in which the decrease in Mal d 1 was enhanced in Golden Delicious by the addition of exogenic CA or EC (Figure 3B). Our data are in agreement with the results reported by Kschonsek et al., who also observed a decline in the quantified Mal d 1 content after incubation (1 h), which was more pronounced in the varieties with a higher PP content.^14^ In a previous correlation analysis conducted by our group, no relationship between the content of monomeric PP and the allergenicity reported for a variety was found.^11^ This might be explained by the formation of browning products in the current experiments. However, during apple consumption, these are negligible.

This effect of the browning products also highlights the importance of the PPO, which accelerates the formation of browning products by catalyzing the oxidation of PP. This is confirmed by the results of Garcia et al. and Gruber et al., who reported an increased decline in IgE-binding affinity of Mal d 1 or r-Pru av 1 in the presence of PP and tyrosinase.^23,24^ Kiewning et al. even proposed that the PPO activity is more important in decreasing the allergenicity of an apple variety than its PP content.^46^ We studied PPO activity in various apple varieties but did not observe significant differences, while high interfruit variations were observed (data not published). However, our experiments with r-Mal d 1.01 demonstrated the importance of PPO, for samples containing CA. In contrast, in the samples containing EC, the r-Mal d 1.01 content decreased regardless of mushroom tyrosinase. This effect might be due to an enhanced oxidation capacity of EC, which is oxidized solely by oxygen.

The four peptide markers analyzed demonstrated a similar decline after incubation (Figure 4). Therefore, it is assumed, that no specific interactions occurred at a particular marker site and that the interactions causing the decline in quantified allergen content affected the entire protein.

### 4.2 Covalent Bond Formation between Cysteine and Epicatechin

The only covalent modification in model systems with r-Mal d 1.01 and EC was observed on the single cysteine at position 107 (Figure 6). None of the other nucleophilic amino acids listed as probable modification sites in the database search were found to be modified. The observed modification at cysteine is in accordance with the results of Unterhauser et al., who also determined that Cys107 in Mal d 1.01 was modified by oxidized CA, while lysine side chains were not affected.^25^ This is probably due to the fact that *o*-quinones react much faster with the thiol group than with an amine or hydroxyl function in amino acid side chains.^47−49^

So far, it is unknown whether a PP bound to the cysteine in Mal d 1.01 influences the IgE binding affinity. However, Cys107 is in proximity to a reported conformational epitope of Mal d 1.01 (Thr10, Ser111, Thr112).^2^ Unterhauser et al. reported that bound CA partially covers these amino acids,^25^ indicating a conceivable effect on IgE binding affinity. This might explain the reduced allergenic potential of the Santana variety, which is characterized by a high proportion of the isoallergen 1.01.^30,37^ Cys107 is specific for isoallergen 1.01, while many other isoallergens do not have any cysteine residue in their amino acid sequence. Exceptions are the isoallergens 1.05, 1.08, and 1.09. However, previous studies have indicated that these isoallergens are not present in significant amounts in apples.^30^ Nevertheless, certain isoforms of the isoallergens 1.03 and 1.06 contain one or more cysteines in their amino acid sequence due to point mutations, and the isoallergen 1.11 contains even four. It has previously been demonstrated that those cysteines are also susceptible to the covalent binding of *o*-quinones.^25^ Therefore, these isoforms will be of particular interest in the future.

### 4.3 Weak Unspecific Interactions of Mal d 1 with Monomeric Polyphenols

Only weak noncovalent interactions were observed by ITC for different flavanols (Table S4, Figure 5). No trend with regard to the molecular weight was obvious. Previous studies, analyzing the binding affinity of flavanols with proteins, also identified a stronger protein affinity for (+)-CAT than for (−)-EC.^18,50^ However, the better binding affinity of (+)-CAT to r-Mal d 1.01 than for the dimers and trimers tested in our experiments is contradictory to the literature data.

Chebib et al. studied interactions between Mal d 1.02 and PP by applying Microscale Thermophoresis.^51^ They determined significantly lower *K*_D_ values for binding of (+)-CAT than (−)-EC with Mal d 1.02 and also reported different *K*_D_ values for (+)-EC and (−)-EC, further illustrating the impact of stereo centers on the binding affinity. By docking studies, they identified hydrogen bonds and hydrophobic interactions as the main factors for Mal d 1.02–flavonoid interactions.^51^ However, it remains unknown if those generally lower *K*_D_ values in the μM range are due to the analytical approach or due to the characteristics of the specific isoallergen (Mal d 1.02).

The weak binding affinity of EC determined by ITC was supported by the limited chemical shift perturbations in the ^1^H–^15^N-HSQC NMR experiments. Generally, ^1^H–^15^N-HSQC NMR demonstrated that binding sites of all PP analyzed were scattered over the whole surface of r-Mal d 1.01 and that they did not bind specifically to a designated binding pocket (Figure 8). In contrast to our results, Unterhauser et al. only identified binding sites of EC in the internal cavity of Mal d 1, while they observed no binding on the outer surface of the allergen.^25^

^1^H–^15^N-HSQC NMR demonstrates that interactions of monomeric PP with r-Mal d 1.01 did not lead to changes in the protein conformation. The lower Δ*S* values in the ITC experiments for PC B2 and PC C1 than for monomeric PP might be explained by aggregation or denaturation of r-Mal d 1.01. These processes are entropically unfavorable since hydrophobic groups, previously localized in hydrophobic areas of the protein, are exposed to the polar solvent. This assumption was supported by ^1^H–^15^N-HSQC NMR for the aggregation of r-Mal d 1.01 with the trimer PC C1 and oxidized EC was demonstrated. This protein aggregation by oligomeric PP might explain the decrease in quantified Mal d 1 with increasing incubation time observed in the model systems containing Goldparmäne and Golden Delicious. Especially the markedly reduced amount of quantified Mal d 1 in the PP-rich variety Goldparmäne and the significant decline in Golden Delicious samples when spiked with CA and EC might be due to the formation of browning products (Figure 3). As the oral phase lasts for less than 2 min, it must be assumed that the aggregation of Mal d 1 by the browning product does not significantly affect the variety-specific allergenicity. However, this effect might explain the better tolerability of apple slices browned before consumption by apple allergy suffers.

### 4.4 No Direct Oxidation of the Tyrosine Side Chain by Polyphenol Oxidase

The PPO can catalyze the hydroxylation of monophenols to *o*-diphenols and the subsequent oxidation of these *o*-diphenols to *o*-quinones. Hence, the tyrosine side chains might be a conceivable substrate for *o*-quinone formation by PPO, leading to protein cross-linking. Garcia et al. hypothesized that this effect might lead to a reduced allergenicity of Mal d 1 due to a time-dependent decline in IgE binding to r-Mal d 1.01 in a model system spiked with mushroom tyrosinase.^24^ Our data do not support this. No significant effect on the marker ITFGEGSQYGYVK, which contains two tyrosines, was observed in the presence of mushroom tyrosinase and 20 min of incubation (Figure 4). Furthermore, the addition of mushroom tyrosinase to r-Mal d 1.01 did not induce signal shifts in ^1^H–^15^N-HSQC NMR experiments (Figure S8), thus excluding cross-linking between oxidized tyrosine side chains and other amino acids. Our results are in accordance with the results of Gruber et al., who observed no effect on the IgE binding affinity of Pru av 1 in the presence of tyrosinase.^23^ Finally, it should be noted, that tyrosine oxidation by PPO under natural conditions is unlikely due to the limited cresolase activity reported for endogenous apple PPO.^52,53^

Combining the results from various analytical approaches, it is demonstrated that only a small proportion of the total Mal d 1 present in apples is released during oral digestion. The rate of Mal d 1 reduction correlated with time, temperature, and the phenolic content, which indicates interactions between oxidized PP and Mal d 1.

Our results reveal unspecific and weak interactions of monomeric and dimeric PP with r-Mal d 1.01, which did not significantly affect the allergen conformation. In addition to these unspecific interactions, a covalent bond formation between EC adducts and the only cysteine in the amino acid sequence of r-Mal d 1.01 was observed. No covalent modifications on other amino acid side chains were identified; however, for apples being rich in isoforms containing cysteine (e.g., Santana) these covalent modifications might shield IgE epitopes. Additionally, r-Mal d 1 aggregated in the presence of phenolics with higher molecular weight, e.g., browning products. For both modes of interactions, *o*-quinone formation is essential. This might explain why oxidized apples are better tolerated by allergic patients than freshly cut fruit. However, due to the short chewing time, it is unlikely that these interactions are the cause for the observed differences in the allergenic potential between traditional and commercial apple varieties

To better understand the correlation between allergenicity, phenolics, and Mal d 1, the interactions of PP with IgEs during the oral phase need to be investigated. Additionally, data about PP and Mal d 1 release from whole apples during chewing are required. Furthermore, deeper knowledge about the impact of cultivation and storage on the decline or increase of isoforms with cysteine is essential.

## Supporting Information Available

Overview of isotopically labeled marker peptides used and their position in the amino acid sequence of Mal d 1 (Figure S1); quantified Mal d 1 content and proportion of release in samples extracted by urea buffer and simulated saliva fluid at the beginning of the oral phase determined by isoallergen-specific markers (Figure S2); Mal d 1 quantified using isoallergen-specific markers at different in vitro oral digestion conditions in the flesh from Golden Delicious and Goldparmäne and effect of supplementation of Golden Delicious with exogenous chlorogenic acid and epicatechin (Figure S3); thermodynamic parameters (stoichiometry *n* = 1) determined by isothermal titration calorimetry for interactions between r-Mal d 1.01 and different flavanols (Figure S4); saturation transfer difference NMR spectrum of r-Mal 1.01 with procyanidin B2 (Figure S5); overlay of ^1^H–^15^N-HSQC NMR spectra of uniform labeled r-Mal d 1.01 in the presence of epicatechin or quercetin-glucoside (Figure S6); partial 1D-^1^H–^15^N-HSQC NMR spectra of r-Mal d 1.01 with chlorogenic acid or quercetin-glucoside (Figure S7); and overlay of ^1^H–^15^N-HSQC NMR spectra of r-Mal d 1.01 with and without mushroom tyrosinase (Figure S8) (PDF)

## Abbreviations Used

^1^H–^15^N-HSQC NMR: ^1^H–^15^N-heteronuclear single quantum coherence NMR spectroscopy
CA: chlorogenic acid
DW: dry weight
EC: epicatechin
ITC: isothermal titration calorimetry
Mal d 1: major allergen 1 in apple
PC: procyanidin
PP: polyphenols
PPO: polyphenol oxidase
Q-glc: quercetin-glucoside
r-Mal d 1.01: recombinant expressed Mal d 1.0108
RT: room temperature
SSF: simulated saliva fluid
STD-NMR: saturation transfer difference NMR
