# Supplementary material for: Multianalytical Approach to Understand Polyphenol-Mal d 1 Interactions to Predict Their Impact on the Allergenic Potential of Apples
Source: J Agric Food Chem. 2024 Jul 11;72(29):16191–203. doi: 10.1021/acs.jafc.4c01555 (PMC11273618; doi:10.1021/acs.jafc.4c01555)
Supplement: Supplementary file 1 — jf4c01555_si_001.pdf [file jf4c01555_si_001.pdf]

Supplemental Material

Multianalytical Approach to Understand Polyphenol–Mal d 1 Interactions to Predict Their Impact on the Allergenic Potential of Apples

Julia A H Kaeswurm[1,2], Birgit Claasen[3]; Pia S Mayer[2]; Maria Buchweitz[1;2;*]

[1] University Hamburg, Department of Chemistry, Institute of Food Chemistry, Martin-Luther-King-Platz 6, 20146 Hamburg, Germany

[2] University Stuttgart, Institute of Biochemistry and Technical Biochemistry, Department of Food Chemistry, Allmandring 5b, 70569 Stuttgart, Germany

[3] University Stuttgart, Institute of Organic Chemistry, Pfaffenwaldring 55, 70569 Stuttgart, Germany

* corresponding author - Maria Buchweitz: E-mail: maria.buchweitz@uni-hamburg.de, Tel.: +49 40 42838-7979

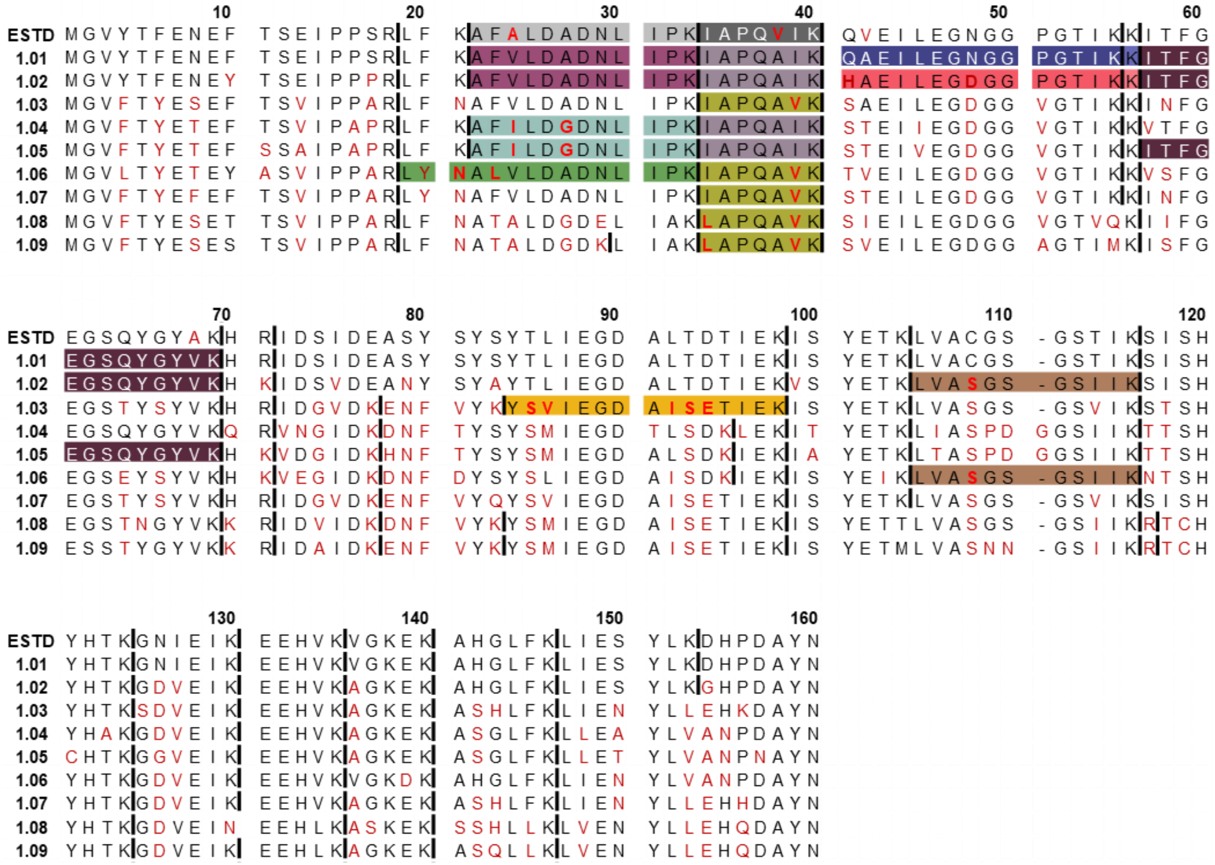

Figure S1: Overview of isotopically labeled marker peptides used and their position in the amino acid sequence of Mal d 1. Previously published in: Kaeswurm et al., New Mass Spectrometric Approach to Quantify the Major Isoallergens of the Apple Allergen Mal d 1, Journal of Agricultural and Food Chemistry. 2022, 70, pp. 11813–11822.

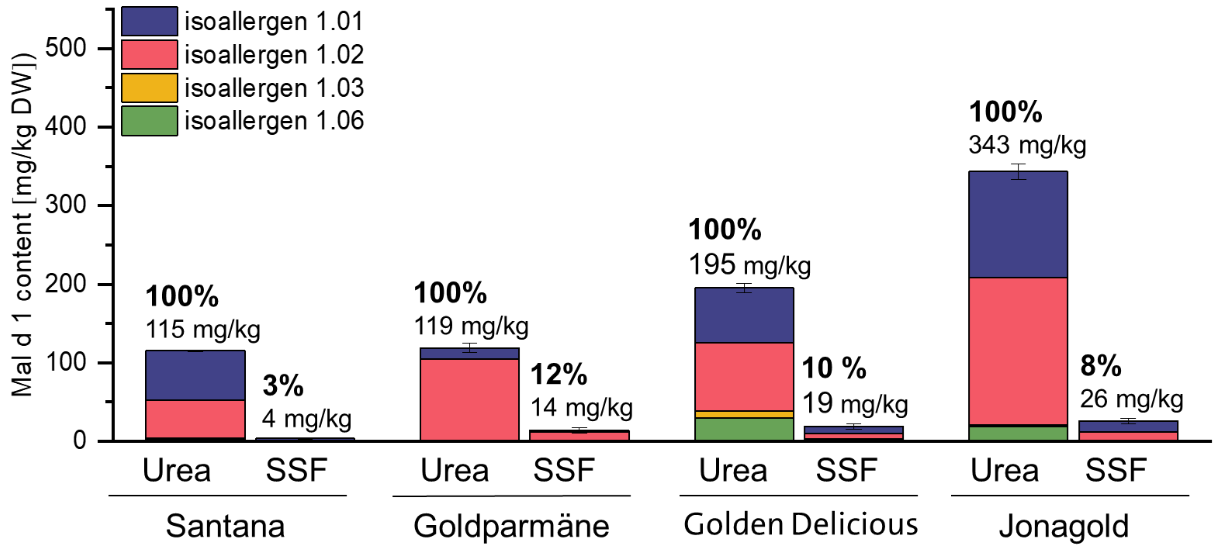

Figure S2: Quantified Mal d 1 content and proportion of release in samples extracted with urea buffer and simulated saliva fluid (SSF) at the beginning of the oral phase determined by isoallergen specific markers.

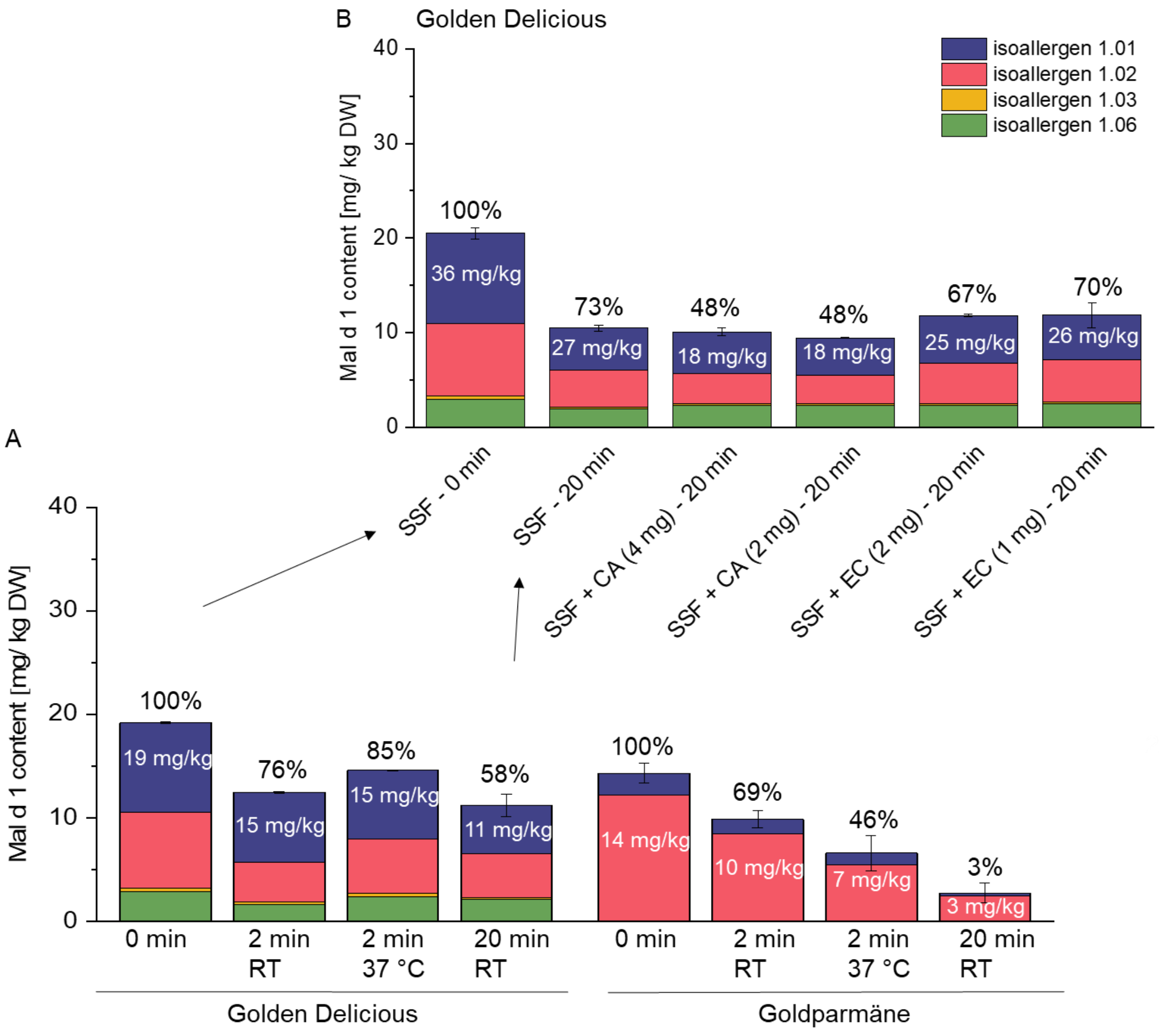

Figure S3: (A) Mal d 1 quantified under different in vitro oral digestion conditions in flesh from Golden Delicious and Goldparmäne using isoallergen specific markers. (B) Effect of supplementation with epicatechin (EC) and chlorogenic acid (CA) in Golden Delicious flesh after incubation for 20 min at room temperature. Mal d 1 quantified is given in white numbers, the proportion (black) is based on the initial (t = 0) release of Mal d 1 in SSF.

Table S4: Thermodynamic parameters (stoichiometry n = 1) determined by isothermal titration calorimetry for interactions between r-Mal d 1.01 and different flavanols.

	Epicatechin[1]	Catechin	Procyanidin B1	Procyanidin B2	Procyanidin C1	oxidized Epicatechin[1,2]
**KD (mM)**	1.4	0.5 ± 0.0	1.2 ± 0.2	0.6 ± 0.0	0.7 ± 0.0	0.7 ± 0.0
**ΔH (kJ/mol)**	-9.4	-13.8 ± 1.8	-10.0 ± 1.7	-16.7 ± 1.1	-15.2 ± 0.2	-12.0 ± 0.2
**ΔG (kJ/mol)**	-16.3	-19.0 ± 0.2	-16.7 ± 0.4	-18.3 ± 0.0	-18.1 ± 0.1	-17.9 ± 0.0
**ΔS (J/mol x K)**	23.3	17.5 ± 6.8	22.7 ± 6.9	5.4 ± 3.4	9.7 ± 0.4	19.8 ± 0.9

[1]Epicatechin and oxidized epicatechin were measured only once. [2]concentration of oxidized epicatechin was determined in the unoxidized stock solution as epicatechin.

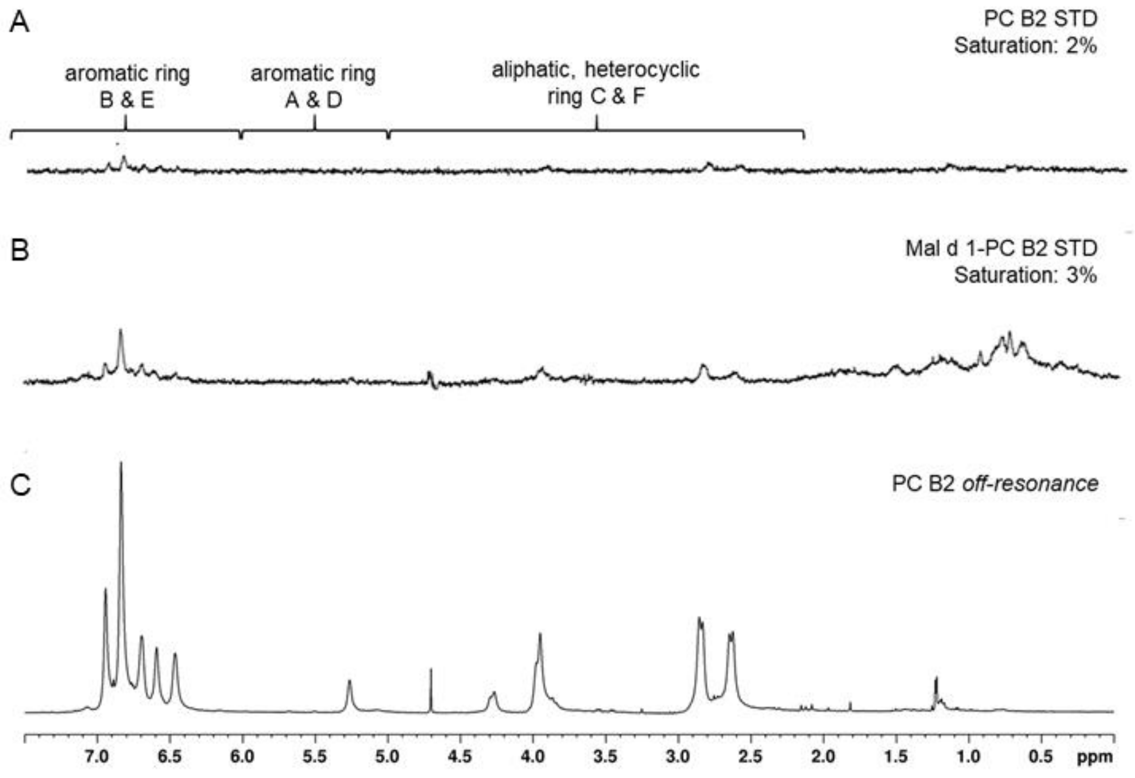

Figure S5: Saturation transfer difference NMR spectrum of 10 µM r-Mal d 1.01 with 1 mM procyanidin PC B2 (B, Mal d 1-PC B2 STD) with the respective off-resonance spectrum (C). The blank spectrum (A) demonstrates the effect of the protein saturation pulse on PC B2.

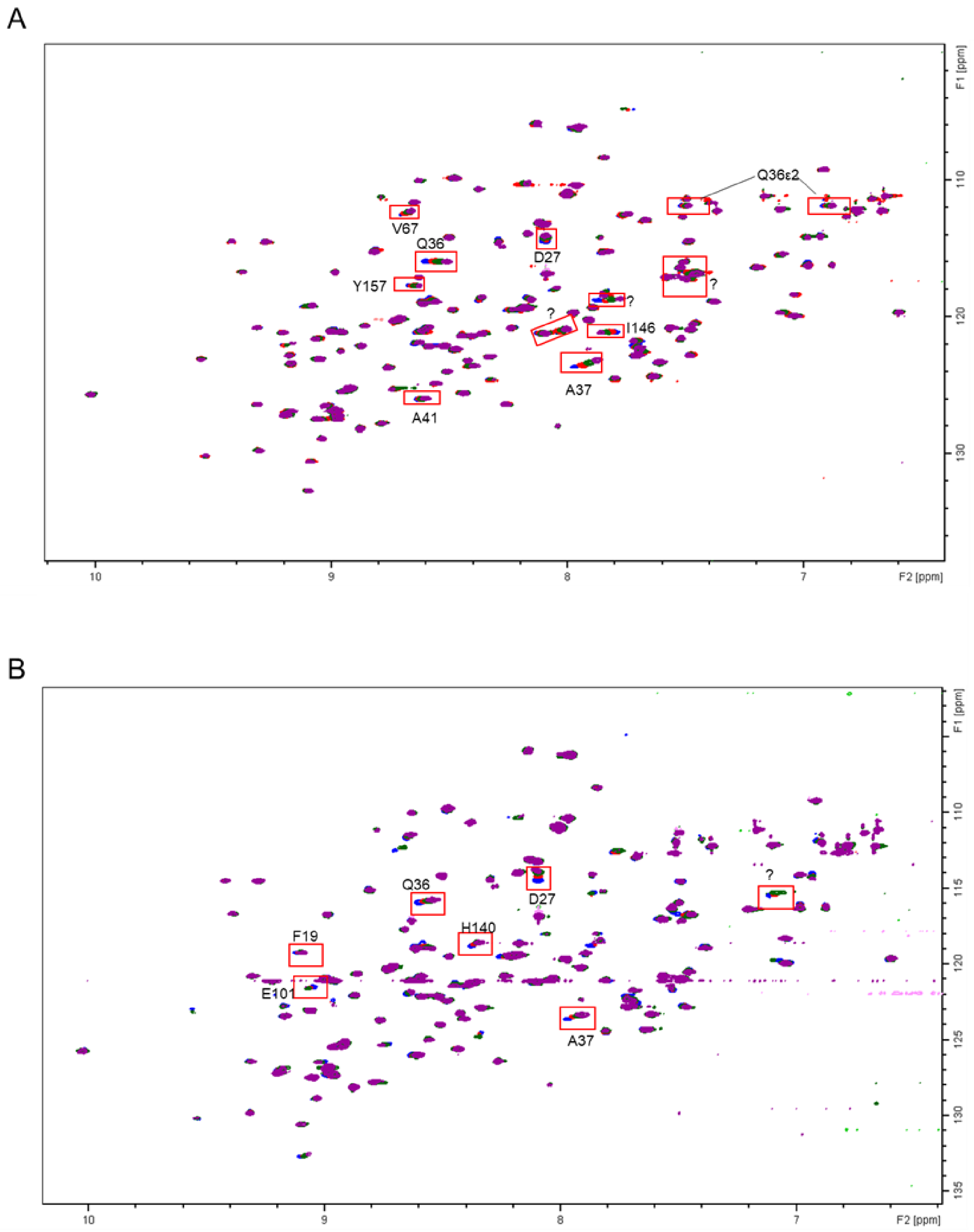

Figure S6: Overlay of ^{1}H-^{15}N-HSQC NMR spectra of the backbone amide protons of 100 µM uniform labeled r-Mal d 1.01 (blue) in the presence of (A) 0.1 mM (red), 1 mM (green) and 2 mM (violet) epicatechin or (B) 0.05 mM (red), 0.10 mM (green) and 0.15 mM (violet) quercetin-3-glucoside.

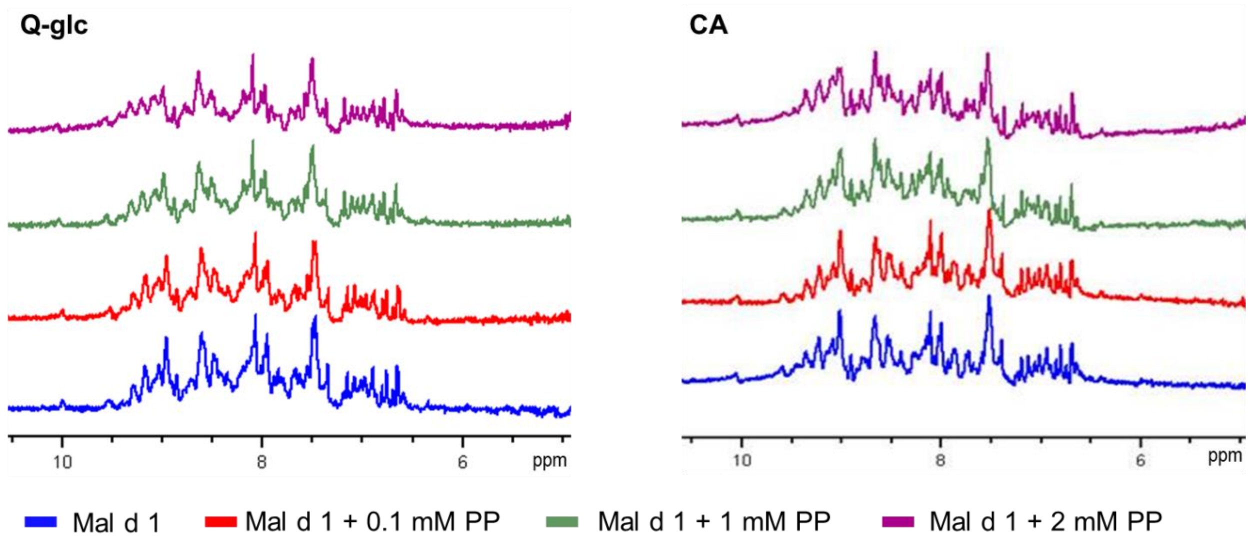

Figure S7: Partial 1D-$^{1}H^{15}N$-HSQC spectra of 100 µM r-Mal d 1.01 and polyphenols (PP) at different concentrations.

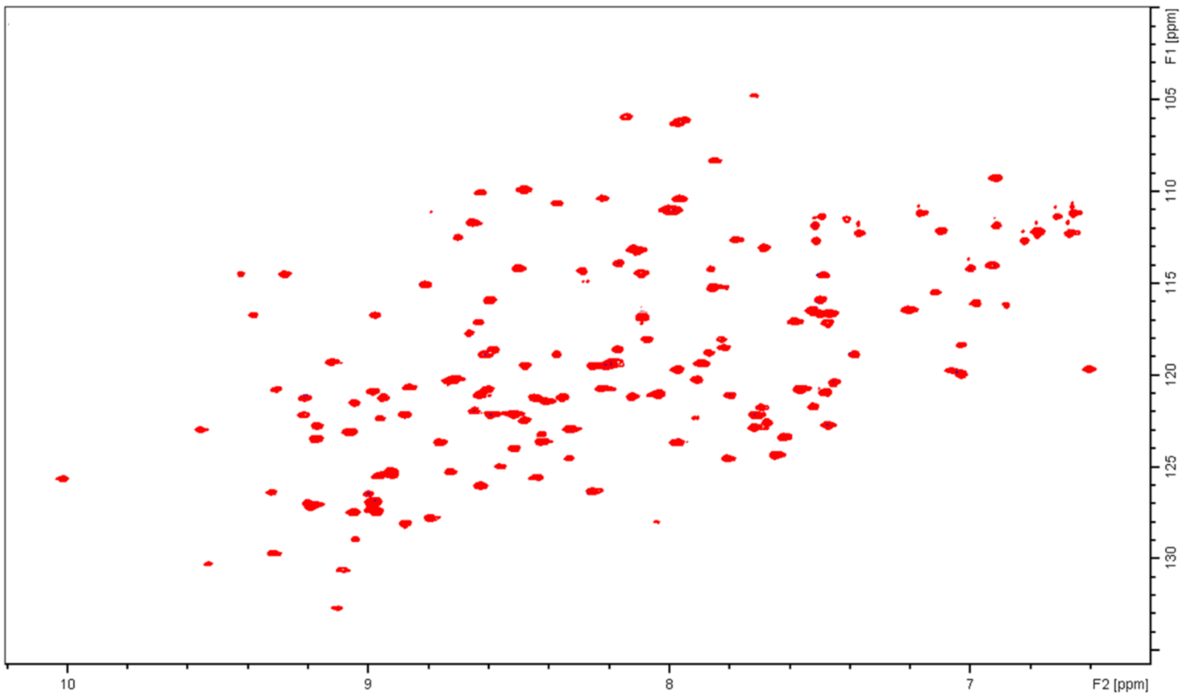

Figure S8: Overlay of ^{1}H-^{15}N-HSQC spectra of the backbone amide protons of 100 µM uniform labeled r-Mal d 1.01 with (red) and without (blue) mushroom tyrosinase addition.
